# Enzymatic Evolution and Longitudinal Recovery in Biotinidase Deficiency: Genotypic and Clinical Insights from the Follow-Up of a Newborn-Screened Cohort in Emilia-Romagna, Italy

**DOI:** 10.3390/metabo15090605

**Published:** 2025-09-10

**Authors:** Rita Ortolano, Soara Menabò, Egidio Candela, Giacomo Biasucci, Elisa Bortolamedi, Giulia Montanari, Alessandro Zuccotti, Umberto Cattini, Marcello Lanari, Federico Baronio

**Affiliations:** 1 Pediatric Unit, IRCCS Azienda Ospedaliero-Universitaria di Bologna, 40138 Bologna, Italy; rita.ortolano@aosp.bo.it (R.O.); elisa.bortolamedi2@unibo.it (E.B.); umberto.cattini@aosp.bo.it (U.C.); marcello.lanari@unibo.it (M.L.); federico.baronio@aosp.bo.it (F.B.); 2 Medical Genetics Unit, IRCCS, Azienda Ospedaliero-Universitaria di Bologna, 40138 Bologna, Italy; soara.menabo@unibo.it; 3 Department of Medical and Surgical Sciences, Alma Mater Studiorum, University of Bologna, Via Massarenti 11, 40138 Bologna, Italy; 4 Pediatrics and Neonatology Unit, Guglielmo da Saliceto Hospital, 29121 Piacenza, Italy; 5 Department of Medicine and Surgery, University of Parma, 43126 Parma, Italy; 6 Specialty School of Pediatrics, Alma Mater Studiorum, University of Bologna, 40126 Bologna, Italy; giulia.montanari22@studio.unibo.it (G.M.); alessandro.zuccotti@studio.unibo.it (A.Z.)

**Keywords:** biotinidase deficiency, newborn screening, BTD enzyme activity, longitudinal follow-up, enzymatic recovery, partial biotinidase deficiency, p.Asp444His variant, genotype–phenotype correlation, biotin therapy

## Abstract

**Background/Objectives:** Biotinidase deficiency (BD) is a treatable autosomal recessive disorder included in many newborn screening (NBS) programs. The importance of early diagnosis and treatment is now well established. However, recent studies are emerging on the possibility of increased enzyme activity with age, an observation that raises questions about the long-term validity of the initial classification of these patients. This study aimed to assess the incidence, genetic and clinical features, and, notably, the longitudinal enzymatic trajectory of BD in a cohort identified by NBS in Emilia-Romagna, Italy, with implications for diagnostic re-evaluation and therapeutic decisions. **Methods:** A retrospective and prospective analysis was conducted on 64 infants recalled after NBS for suspected BD between 2016 and 2020. Biochemical, molecular, and clinical data were collected, and biotinidase (BTD) activity was monitored longitudinally. Affected individuals were supplemented with biotin and followed clinically for at least 5 years. **Results:** Thirty-one patients were diagnosed with BD (30 partial, 1 profound; incidence 1:5448). A significant and sustained increase in BTD activity was observed from diagnosis through early childhood (*p* < 0.001 up to 60 months), particularly among patients carrying the p.Asp444His variant. This enzymatic trend suggests a potential remodulation of biochemical classification over time. Genotype–phenotype concordance was high (92%), and clinical outcomes were favorable across the cohort. **Conclusions:** This study provides new evidence that BTD activity in patients with BD increases progressively, supporting the concept of age-dependent enzyme recovery. Our results support the need for systematic re-evaluation of diagnosis and treatment, especially at 12 months of age, and particularly in patients with evidence of partial activity deficiency and the p.Asp444His mutation.

## 1. Introduction

Biotinidase deficiency (BD, OMIM: 253260) is an autosomal recessive metabolic disease, first described in 1981 [1]. This condition is characterized by a congenital defect in biotin recycling, associated with secondary alterations in the metabolism of amino acids, carbohydrates, and fatty acids [2,3]. Mutations of the BTD gene result in absent or reduced activity of the biotinidase enzyme involved in the biotin cycle [4], causing, respectively, a profound deficit if the residual enzymatic activity (AER) is <10% or a partial deficit if AER is between 10% and 30% [5,6].

The incidence of BD worldwide is between 1:40,000 and 1:60,000 newborns, with considerable variability between countries [7] and a higher incidence in countries with a high rate of consanguinity, such as Turkey and Saudi Arabia [3,8].

In Italy, particularly in the Piemonte and Valle d’Aosta regions, data from the NBS program collected between January 1987 and December 2023 indicate a combined incidence of profound and partial deficiency that is consistent with the global rate, approximately 1 in 25,000 [9]. Another recent Italian study found that the incidence of BD identified by NBS in the regions of Tuscany and Umbria was ten times higher (1:6300) than the global average. This data suggests that the pathology may have often been underdiagnosed, with an incidence detected in retrospective clinical studies being erroneously lower. The high incidence observed in the study is likely related to the high frequency of specific mutations in the Italian population Another recent Italian study reported that the incidence of BD detected through NBS in the regions of Tuscany and Umbria was nearly tenfold higher (1:6300) than the global average. These findings suggest that the disorder has likely been underdiagnosed, as incidence estimates derived from retrospective clinical studies appear to have been erroneously lower. The elevated incidence observed in this study is plausibly attributable to the high prevalence of specific mutations within the Italian population [10].

The correlation between genotype and biochemical and clinical phenotype remains unclear [5], even for genotypes that cause profound or partial deficits. The clinical manifestations related to this pathology are rather heterogeneous; subjects affected by profound deficit can develop complications such as convulsions, hypotonia, ataxia, developmental delay, visual problems, neurosensory deafness, alopecia, skin rash, and infections. In patients with partial deficiency, however, if subjected to stressful conditions, hypotonia, skin rash, and hair loss may be observed [11]. A systematic review of the literature published in 2023 reports the presence of symptoms in 482 out of 1113 patients described (equivalent to 43.3%), of whom 82.2% had more than one system/organ affected at the time of diagnosis [12].

Early treatment with biotin for this condition can prevent the onset of symptoms. Therefore, BD appears to be an optimal model of pathology to be subjected to NBS [13,14]. In addition to adequate treatment, periodic follow-up evaluations are recommended for these patients to identify any early signs or symptoms of the pathology [5].

A recently published paper by Forny et al. [15] introduces the possibility of re-evaluating the diagnosis based on an age-related, progressive increase in BTD enzyme activity, particularly after the age of five years, at which point retesting of patients could be recommended.

The purpose of this study is to provide a critical analysis of the clinical experience regarding BD screening in children born from January 2016 to December 2020 in the Emilia-Romagna Region, with a specific focus on the long-term follow-up of these patients. The incidence of the disease in Emilia-Romagna will be evaluated by comparing it with that described in other national and international settings. The clinical and genetic investigation data obtained from patients and parents will be described. Data from a minimum of 5 years of follow-up for the 31 patients with BD who attend this center will be described. Ultimately, we sought to comprehensively evaluate the age-related trajectory of BTD enzyme activity within our patient cohort. The aim of this study is to critically analyze the clinical experience with BD screening in children born between January 2016 and December 2020 in the Emilia-Romagna Region, with a particular emphasis on long-term follow-up. The incidence of the disorder in Emilia-Romagna will be assessed in comparison with that reported in other national and international contexts. Clinical and genetic data from patients and their parents will be presented, together with findings from a minimum of five years of follow-up in 31 BD patients monitored at this center. Finally, we seek to provide a comprehensive evaluation of the age-related trajectory of BTD enzyme activity within this cohort and the need for retesting of patients as recommended by Forny et al [15].

## 2. Materials and Methods

We conducted a retrospective analysis of patients affected by BD identified from January 2016 to December 2020 in the Emilia-Romagna Region, as part of the NBS Regional Program of Bologna. We have obtained the nullaosta to conduct this non-profit study by the Ethics Committee of the Emilia Centro Vast Area of the Emilia-Romagna Region at the Sant’Orsola University Hospital in Bologna (number EC 113/2021/Oss/AOUBo).

### 2.1. Screening Protocol and Diagnostic Confirmation Methods

In our Region, the NBS of BD was introduced in January 2016. The BTD enzyme activity is determined on newborn dried blood spots (DBS), collected between 48 and 72 h of life. BTD testing is not performed as a stand-alone program but is included as one of the conditions within the expanded newborn screening panel, which currently covers more than 50 metabolic, immunological, neurological, and endocrine disorders. The enzyme activity on DBS is performed semi-quantitatively, measured using the GSPNeonatalBiotinidase kit (PerkinElmer, Wallac Oy, Turku, Finland); this assay combines an enzyme reaction with a solid-phase time-resolved immunofluorescence assay. If the first NBS was below the cut-off, we would distinguish low or high risk of recall according to the recall thresholds.

From January 2016 to December 2019, we employed a single high-risk recall threshold of 107 U/dL (corresponding to 50% of AER, calculated based on the neonatal population of Emilia-Romagna) for DBS. From January 2020, based on the review of our BTD recalls and confirmations, we established that a low risk exists if BTD is at the first DBS results between 65 and 85 U/dL (corresponding to 10–30% of AER), and a high risk exists if the first or second DBS results are <65 U/dL.

All patients recalled from January 2016 to December 2019 for AER <107 U/dL repeated DBS, and if it returned positive (<107 U/dL), molecular analysis of the BTD gene was performed. In patients recalled from January 2020, molecular analysis of the BTD gene was performed immediately in newborns with a high risk of recall (AER < 65 U/dL) or after the second pathological DBS in newborns with a low risk of recall (AER: 66–85 U/dL). Biotinidase enzymatic activity was expressed either in units per deciliter (U/dL) or as a percentage of mean normal activity. For conversion purposes, reference values were defined as follows: 21.6 U/dL corresponds to 10% of mean normal activity, 65 U/dL corresponds to 30% and 108.4 U/dL corresponds to 50%. The loss of BTD activity was considered profound if <10% of normal activity, partial when between 10 and 30% and mild reduction if between 30 and 50%. Between January 2016 and December 2019, all newborns recalled for AER < 107 U/dL underwent repeat DBS testing; if the result remained abnormal (<107 U/dL), molecular analysis of the BTD gene was performed. From January 2020 onward, molecular analysis was conducted directly in newborns at high risk of recall (AER < 65 U/dL) or following a second abnormal DBS in those at lower risk of recall (AER 66–85 U/dL). Biotinidase enzymatic activity was expressed either in units per deciliter (U/dL) or as a percentage of mean normal activity. For conversion purposes, reference values were defined as follows: 21.6 U/dL = 10% of mean normal activity, 65 U/dL = 30%, and 108.4 U/dL = 50%. Loss of BTD activity was classified as profound (<10% of normal activity), partial (10–30%), or mild (30–50%).

The molecular analysis of the BTD gene was performed on DNA extracted from peripheral blood leukocytes following a standard procedure. The coding sequence of the BTD gene, including exon–intron boundaries, was directly sequenced using a standard Sanger procedure.

Sequence variants were designated according to Human Genome Variation Society recommendations (https://www.hgvs.org/content/guidelines, accessed on 5 June 2025) using the reference sequence GenBankNM_000060.4.

Only the variants classified as pathogenic, likely pathogenic, or of uncertain significance (VUS), according to the American College of Medical Genetics and Genomics (ACMG) standards [16], were taken into account; different platforms, databases, and archives are used for this purpose: Varsome [17], ClinVar [18], LOVD [19], and Franklin by Genoox (https://franklin.genoox.com, accessed on 6 June 2025). The classification of all variants was updated as of June 2025.

For each variant identified, databases available online have been used to verify if variants were already reported in the literature, such as Pubmed (https://pubmed.ncbi.nlm.nih.gov/, accessed on 7 June 2025), ClinVar [18], and Human Genetic Mutation Database (HGMD; https://www.hgmd.cf.ac.uk/, accessed on 5 June 2025), and to assess the severity in terms of residual enzymatic activity.

To assess the concordance between biochemical phenotype and genotype, patients were classified into three groups according to their phenotypic profile: group A, patients with profound deficiency (AER < 10%); group B, patients with partial deficiency (AER 10–30%); and group C, non-pathological patients (AER > 30%).

Patients affected by BD (groups A and B) underwent clinical and biochemical follow-up, including evaluation of hepato-renal function, AER on DBS, audiometry, ophthalmologic assessment, and pediatric examination to monitor skin manifestations, growth, and neurological development. In addition, biotin supplementation was initiated in all affected patients, at a dosage of 10 mg/day for profound BD and 5 mg/day for partial BD.

During follow-up, the natural progression of BTD enzyme activity was monitored through serial DBS measurements in patients with BD (groups A and B).

### 2.2. Statistical Analysis

To assess the evolution of BTD enzymatic activity over time, we conducted pairwise comparisons between values at diagnostic confirmation (t0.5 m, approximately 2 weeks of age) and subsequent follow-up time points (t3, t12, t36, t60 months). The Wilcoxon signed-rank test was applied to paired observations, and *p*-values were adjusted for multiple comparisons using the Bonferroni correction.

## 3. Results

Among 168,889 newborns screened in the Emilia-Romagna Region from January 2016 to December 2020, 64 were recalled to repeat DBS and underwent BTD genetic analysis. Of these, 31 were diagnosed with BD, corresponding to an overall incidence of 1:5448 newborns (1:5629 for partial BD and 1:168,889 for profound BD).

Table 1 shows the results of genetic analysis of all 64 patients, with relative biotinidase activity measured at diagnosis, the type of BD predicted based on genotype and enzyme activity, and classification into groups A, B, and C.

Group A (patients affected by profound deficit) consisted of a single patient (**ID 1**), who presented the known severe pathogenic variant p.Gln456His in homozygosis. The patient, therefore, has a biochemical phenotype that reflects the severity of the genotype.

Group B (patients with partial deficiency) included 30 patients with an AER on the second spot between 41 U/dL (**ID 4**) and 66.3 U/dL (**ID 9**), mean 53.67 ± 6.91 U/dL. All 30 patients were found to be compound heterozygotes for the known p.Asp444His variant associated with other variants: in 9 patients with the severe variant p.Gln456His, in 5 cases with complex allele p.Asp444His;p.Ala171Thr and in another 5 cases with p.Cys33PhefsTer36.

In three patients from Group B, three previously unreported variants were identified in association with the known p.Asp444His variant. Specifically, p.Glu436AlafsTer8 was identified in a patient with an AER of 23.5% (**ID 4**), p.Gly238AlafsTer36 in a patient with an AER of 23.8% (**ID 31**), and p.Met98_Ala100delinsIle in a patient with an AER of 22.6% (**ID 30**). Given the diminished AER observed in these patients, it is plausible that these novel variants exert a substantial impact on BTD function.

The other eight patients were compound heterozygous with different variants already reported in the literature. In all 30 patients, the biochemical phenotype agreed with the expected genotypic group reflecting the presence of the p.Asp444His variant in association with other allelic variants, which determine the phenotype.

Group C (unaffected patients) included 33 individuals with a second-spot AER ranging from 67.7 U/dL (**ID 60**) to 188.19 U/dL (**ID 55**), with a mean value of 88.53 ± 21.29 U/dL. The most frequent genotype in this group was homozygosity for p.Asp444His, which also represented the most common genotype in our overall cohort (19/64). Ten patients carried the p.Asp444His variant in compound heterozygosity with different additional mutations. Among these, three individuals (**ID 57, 58, 59**) harbored the p.Pro133Leu variant on the second allele, a change not previously reported in the literature and absent from group B. Although rare, the p.Pro133Leu variant appears to be associated with only a mild reduction in enzymatic activity, suggesting partially preserved BTD function. Its recurrence in multiple patients within our cohort underscores its potential clinical relevance and warrants further functional characterization.

The novel variants identified in Group C included p.Pro133Leu, observed in three patients in compound heterozygosity with p.Asp444His and p.Gly293Ser, and p.Phe361Ser, detected in compound heterozygosity in a single patient. All were missense variants classified as either likely pathogenic or of uncertain significance (VUS). Their impact on residual enzymatic activity remains unknown but is presumed not to result in profound deficiency.

Two patients (**ID 51 and 52**) carry the compound allele p.Asp444His;p.Ala171Thr [20], a genotype that, in contrast, is well represented in group B. However, these two patients do not exhibit a partial deficiency, similarly to three other patients (**ID 53, 54, and 55**) who harbor severe variants on the second allele (p.Arg157His, p.Pro497Ser, and p.Cys33PhefsTer36, respectively)

Only 4 cases do not include the p.Asp444His in their genotypes: 2 patients (**ID 62, 63**) are homozygous for the p.His323Arg, one patient (**ID 61**) is compound heterozygous for two novel VUS variants, and one (**ID 64**) is a simple heterozygote for the severe variant p.Gln456His.

The genotype-phenotype concordance is therefore 84.8% in group C (5/33 discordant patients).

Considering the entire study population (group A, B and C), the genotype-phenotype concordance is 92% (5/64 discordant patients)

The molecular analysis of the BTD gene revealed a total of 20 different variants (listed in Table 2), distributed across 23 distinct genotypes.

All the variants, except two, are classified as pathogenic or likely pathogenic. Only patient **ID 61** has two VUS variants, p.Gly293Ser and p.Phe361Ser, not reported in the literature.

### Follow-Up

Table 3 reports the laboratory and clinical follow-up data of the 31 patients diagnosed with BD.

Enzymatic activity was assessed at various and available follow-up time points using dried blood spots (DBS) for each patient. To facilitate interpretation and longitudinal monitoring, patients were categorized into three groups based on a traffic light color system, reflecting distinct patterns of enzyme activity:

- **Red group:** Patients whose BTD enzymatic activity remained persistently below 30% at all time points. These individuals showed no evidence of biochemical recovery during follow-up.
- **Yellow group:** Patients with fluctuating enzymatic activity, characterized by intermittent values above and below the 30% threshold. The timing of the first observed increase above 30% is documented in the column “Age at BTD Enzyme Activity Recovery.”
- **Green group:** Patients whose enzymatic activity increased above 30% and remained consistently above this threshold from a given follow-up point onward, indicating a sustained biochemical recovery.

This classification system enables a standardized, visual representation of enzymatic trajectory over time, and may support future decisions regarding therapeutic management and follow-up intensity.

Three patients are classified as red, 13 as yellow and 15 as green (Table 3).

Most patients demonstrated favorable clinical outcomes. At the time of the last evaluation, 19 out of 31 patients were reported as healthy, with no significant complications or sequelae. A minority exhibited mild and non-progressive findings, including dermatologic signs (e.g., café-au-lait macules, hypertrichosis), ophthalmologic conditions (e.g., alternating esotropia), recurrent asthma-like bronchitis, and mild language delays managed with speech therapy. These findings were observed across both the “yellow” (characterized by fluctuating enzyme levels) and “green” (characterized by stable levels above 65 U/dL) groups.

Three patients discontinued follow-up during the COVID-19 pandemic, likely due to difficulties in arranging outpatient visits during that specific phase.

Two (**ID 21** and **ID 23**) were followed for 3 years and one (**ID 22**) for 12 months. Their biotinidase activity was collected and analyzed as long as possible. **ID 21** and **ID 22** were siblings and both showed significant increase in biotinidase activity by 12 months; conversely **ID 23** showed fluctuant enzyme activity at the last available follow-up time point (3 years).

At the time of the last clinical evaluation, all patients except one were receiving 5 mg/day of oral biotin supplementation. The only patient treated with 10 mg/day was the single case classified as having profound BD, reflecting a tailored therapeutic approach based on biochemical severity. No adverse events related to biotin were reported, and none of the patients discontinued therapy on their own.

**Figure 1:**
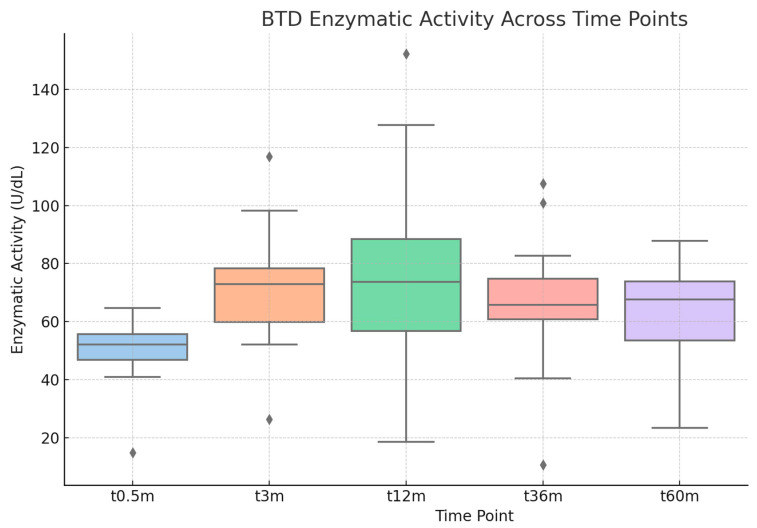
Boxplot of enzyme activity (on the *y*-axis) measured at various time points (on the *x*-axis) in patients of group A and B. t05 m = 2 weeks of age, t3 m = 3 months of age, t12 m = 12 months of age, t36 m = 36 months of age, t60 m = 60 months of age.

Furthermore, Figure 1 shows a box plot of BTD enzyme activity measured at different time points: 2 weeks (t0.5 m, period corresponding to diagnostic confirmation), 3 months (t3 m), 12 months (t12 m), 36 months (t36 m), and 60 months of age (t60 m). The distribution of enzyme activity shows a general upward trend from birth to early childhood. In detail, we observed a progressive increase in values between the 3-month and 12-month check-ups, with stabilization around the first year of life. Another interesting finding is the dispersion of values, with the interquartile range decreasing between t12m and t60 m, suggesting less variability in BTD enzyme activity at this stage and, therefore, greater stabilization as the months progress.

A highly significant increase in BTD activity was observed between t0.5 m and all subsequent time points. In particular, the difference was strongest within the first year of life, both at 3 months (*p* < 0.001) and 12 months (*p* < 0.001), but statistical significance remained evident at later time points as well, including 36 months and 60 months.

The maximum average enzyme activity value was observed at 12 months, with an average of 75.32 U/dL ± 26.9 U/dL.

Overall, these results demonstrate a significant and sustained increase in BTD enzymatic activity from diagnosis through early childhood, with a potential plateau beyond 5 years of age.

## 4. Discussion

Our study indicates that the overall incidence of BD in the Emilia-Romagna Region during the first five years after the introduction of newborn screening for this condition was 1 in 5448. Although this incidence is markedly higher than the globally estimated rate [6], it is consistent with findings from other Italian studies [10,30,31] and likely reflects a higher prevalence of BTD gene mutations in the European population [32].

In Italy BTD testing is not performed as a stand-alone program but is integrated into the expanded newborn screening panel. This integration makes the additional cost per newborn negligible, while the clinical and socioeconomic benefits are substantial, as early detection and treatment prevent irreversible neurological damage. The incidence that we observed further supports the inclusion of BD in expanded NBS panels wherever an organized infrastructure is in place. From a public health perspective, the minimal added cost is largely outweighed by the benefits of preventing possible irreversible neurological damage and lifelong disability.

Consistent with previously published data, the most frequently observed variant in our cohort was p.Asp444His, detected in 59 of 64 patients. This variant has an allelic frequency of 0.039 in the general population [28] and, when present in heterozygosity as a single variant, is associated with an AER of approximately 75% [6]. In homozygosity, it typically results in a residual enzymatic activity of 45–50% [11]. Consistent with these findings, the homozygous p.[Asp444His]; [Asp444His] genotype was the most frequent in group C (19 patients) and was associated with residual enzymatic activity ranging from 31.5% to 48.3%.

It is well established that the presence of the p.Asp444His variant in compound heterozygosity with a severe mutation accounts for the vast majority of partial BD cases [28]. Accordingly, 100% of our patients with partial deficiency (group B) were found to be compound heterozygotes for p.Asp444His and another pathogenic variant with a predicted severe impact on enzymatic activity.

The p.Asp444His variant has been compared, in terms of its effect on the biochemical phenotype, to the Duarte variant of galactosemia; the latter also determines an enzymatic activity of 75% [3] in heterozygosis, while in compound heterozygosis with the variant of classical galactosemia, an AER of 25% [28].

Among the variants known to cause severe BD, the most frequently reported are p.Cys33PhefsTer36, p.Gln456His, p.Arg538Cys, and the complex allele p.Asp444His;p.Ala171Thr [5]. All of these were also identified in combination with p.Asp44His in our cohort of patients with partial deficiency (group B).

In group B, we identified three allelic variants that had never been reported before: two frameshifts (p.Gly238AlafsTer36, p.Glu436AlafsTer8) and one *delins* variant (p.Met98_Ale100delinsIle). It is well established that the partial deficiency phenotype results from the combination of p.Asp444His with a severe mutation that reduces residual enzymatic activity to below 10% [33]. Therefore, it is plausible that the three novel variants identified in our patients—who showed residual enzymatic activity between 22.6% and 23.8%—have a significant impact on enzyme function.

To date, over 200 mutations in the BTD gene have been identified [34], however the genotype-phenotype correlation remains not fully defined.

Our data show a genotype-biochemical phenotype concordance of 92%, which reaches 100% in the group with partial deficiency. The only patient with profound BD presents the known pathogenic variant p.Gln456His in homozygosis, as described by Norrgard et al. [35] as pathogenic and severe.

A higher rate of genotype–phenotype discordance was observed in patients with an AER above 30% (classified in group C). Specifically, we found only mildly reduced residual enzymatic activity in five patients with genotypes typically associated with partial deficiency (compound heterozygotes for the p.Asp444His variant and one of the following known severe mutations: p.Asp444His;p.Ala171Thr, p.Arg157His, p.Pro497Ser, or p.Cys33PhefsTer36).

The incomplete correspondence between genotype and phenotype observed within our research is widely acknowledged in the literature for BD [5] and may be due to epigenetic factors that influence the expression of allelic variants or to other factors yet to be identified.

Additionally, the ACMG classification of the variant is not linear, and different tools yield varying interpretations of pathogenicity.

With regard to therapeutic management, all patients with partial BD continued supplementation with 5 mg of biotin daily. In contrast, the patient diagnosed with profound deficiency received a higher dose (10 mg/day), in accordance with current recommendations for the severe form [11]. It is noteworthy that biotin therapy exhibits a favorable safety profile, even with long-term use, as evidenced by the absence of side effects.

Our clinical outcomes were notably positive: over 60% of patients were reported as entirely healthy at their last follow-up, with only a minority exhibiting mild, non-progressive signs such as skin manifestations, esotropia, or language delay. These findings align with previous studies, which demonstrate that individuals with partial BD who are treated typically remain asymptomatic or exhibit minimal manifestations [26]. Such a favourable clinical outcome leads us to believe that BD is an ideal condition for neonatal screening, both due to the absence of early acute neonatal decompensation [36] and the relative reliability of direct measurement of enzyme activity, compared to indirect markers such as methionine in homocystinuria [37], which are more controversial.

Notably, in a recent systematic review of the literature, the most frequent clinical presentation of BD (reported in 48.1% of symptomatic patients) was a combination of cutaneous manifestations and neurological involvement [12]. The presence of such features in a few patients—most of whom had partial BD—may be coincidental or reflect common conditions in the general pediatric population, suggesting a multifactorial etiology rather than a direct consequence of BD itself.

The absence of symptoms in patients diagnosed and treated with biotin at such an early stage of life does not allow us to determine whether the positive outcome is attributable to early therapy or whether it would have occurred regardless of our therapeutic intervention. The implementation of prospective, double-blind, randomized controlled trials (RCTs) involving genotype- and enzyme activity-matched patients, randomized to receive either biotin supplementation or a placebo, would represent the gold standard for ascertaining the actual causal impact of early therapeutic intervention on clinical outcomes. However, the possibility of conducting such a trial seems to be burdened by critical ethical considerations, which must be taken into account when dealing with a metabolic disorder that is currently treatable and capable of causing significant clinical consequences. Alternatively, rigorously designed observational studies, such as matched cohort designs, could be a viable and ethically acceptable strategy for understanding the impact of treatment in real-world settings.

Another notable observation is the interruption of follow-up for the three patients during the COVID-19 pandemic. This data supports the theory, already evident in the early months of the pandemic, that patients with chronic conditions, especially rare ones concentrated in a few centers in the Region, which require significant travel by their families, have often been the most disadvantaged in this particular historical period [38].

Our results reinforce the importance of systematic longitudinal monitoring in patients diagnosed with BD, particularly within the first year of life.

The significant increase in enzymatic activity observed between the time of diagnostic confirmation (t0.5 m) and all subsequent time points up to 60 months, with the most pronounced rise observed in the first 12 months of life (*p* < 0.001 for all comparisons up to t60 m) and the peak of activity at 12 months (mean activity 75.32 U/dL ± 26.9 U/dL), strongly suggests that this timeline represents a key turning point in the biochemical profile of many patients.

Forny et al. [15] demonstrated the possibility of an age-related increase in BTD enzymatic activity, which could influence therapeutic strategies for individuals with BD. In this study, the improvement of approximately 75% of cases was correlated with the presence of the p.Asp444His variant on at least one allele, suggesting a pivotal role for this mutation in enzymatic recovery. In our cohort, all patients who exhibited an increase in enzymatic activity had at least one allele with the p.Asp444His variant, which in two cases was found in *cis* with an additional mutation on the same allele. Our findings therefore further support the central role of the p.Asp444His variant in driving enzymatic recovery and suggest that diagnostic re-evaluation may be appropriate in patients harboring this mutation, particularly around the age of 12 months. However, the presence of the p.Asp444His variant alone does not determine the possibility of recovering enzymatic activity. In fact, patients **ID12** and **ID27** never showed enzymatic activity greater than 30% during follow-up.

The possibility that enzyme activity may increase with age is a phenomenon that has not yet been explained, although it has already been observed in other enzymes. In animal studies, such as in aged rats, sphingomyelinase and ceramidase, two enzymes involved in sphingolipid metabolism, have been shown to exhibit progressively increasing enzymatic activity over time [39].

Given these dynamics, re-evaluation at 12 months emerges as a critical clinical milestone. It allows for an objective reassessment of the enzymatic trajectory and may serve as the most appropriate time point to differentiate between transient or borderline phenotypes and those with stable enzymatic insufficiency.

### Limitations

This study has several limitations. Firstly, the study is retrospective, which may induce a known patient selection bias and limit the ability to investigate causes, as the sample was not randomly recruited but derived from available clinical records. Furthermore, BTD enzyme activity was assessed exclusively on DBS, without confirmatory testing on alternative biological matrices.

Biotinidase activity on DBS could be underestimated due to improper storage and excessive heat or humidity exposure.

As known, the enzymatic activity of BTD is unstable and fluctuates for various reasons, including preanalytical variables (such as sample storage temperature and transport logistics), which can significantly influence the enzymatic value obtained. Seasonality, with its associated changes in ambient temperatures and sample storage conditions, could also be a confounding factor in the evaluation of enzyme activity and genotype-phenotype correlation.

Furthermore, three patients were lost during follow-up and could not be further evaluated. For this reason only partially available data were analyzed. Neverthless, we decided to include their data in order to consider their peculiar genetic variants. Moreover, these patients had shown significant and stable increase in enzymatic activity during the first 12 months of follow-up (**ID 21** from 27.28% to 37.86%; **ID 22** from 24.74% to 45.35%).

Another significant limitation is that our cohort is relatively small, which reduces statistical power and limits the generalizability of the results. Finally, this is a pilot study; however, a prospective phase is currently underway, which will provide additional longitudinal data to support and refine the present observations.

## 5. Conclusions

The findings of this study confirmed that the incidence of BD is higher than the global average but comparable to that reported in other studies of the Italian population [10,30]. This may be related to the recurrent presence of specific pathogenic variants within the Italian population and/or to the higher sensitivity of the NBS system compared with that of other countries.

Furthermore, our data confirm that it is not always possible to establish a correlation between genotype and phenotype, although in our cohort the level of concordance was very high. Such discrepancies may depend on interfering factors (e.g., variable expressivity of certain genotypes, epigenetic influences), but further studies are warranted to clarify these observations.

Since January 2020, our regional NBS program for BD has been refined by lowering cut-off values and implementing a multi-threshold recall system, leading to increased specificity. The system appears robust and reliable, as supported by the absence of complications observed in our patients.

It is significant that most of our patients remained asymptomatic or showed mild, non-progressive symptoms. In patients carrying the p.Asp444His variant, a substantial increase in enzyme activity was observed with advancing age, particularly evident in the first year of life. We therefore consider it crucial to emphasize the importance of progressive diagnostic re-evaluation over time, particularly at one year of age, in order to individualize follow-up strategies. According to our experience, the major recovery of enzymatic activity occurs by 12 months of age. Therefore, we suggest re-assessing biotinidase activity at 12 months and subsequently every one to two years, at least until the age of 5 years. Beyond this age, the utility of further re-assessments should be investigated.

Our study builds on and expands the study by Forny et al. [15], including the consideration of additional genotypes, the use of a different analytical method, and additional time time points for determination of BTD enzymatic activity.

Finally, larger and prospective studies are needed to better understand the natural history of BTD and to optimize long-term management, including the development of shared guidelines, particularly in light of the challenges posed by rare disease research and the ethical constraints of trial design.

## Figures and Tables

**Table 1:** Biochemical and molecular profile of patients with reduced biotinidase activity (*n* = 64).

Patient ID	Allele 1	Allele 2	Expected Type of BD According to Genotype	Type of BD According to Enzyme Activity	BTD Activity U/dL at Diagnosis (%)	Group
1	p.Gln456His	p.Gln456His	Profound	Profound	15.0 (6.9)	A
2	p.Asp444His	p.Gln456His	Partial	Partial	54.6 (25.2)	B
3	p.Asp444His	p.Gln456His	Partial	Partial	64.7 (29.8)	B
4	p.Asp444His	p.Gln456His	Partial	Partial	41.0 (18.9)	B
5	p.Asp444His	p.Gln456His	Partial	Partial	55.5 (25.6)	B
6	p.Asp444His	p.Gln456His	Partial	Partial	53.5 (24.7)	B
7	p.Asp444His	p.Gln456His	Partial	Partial	47.5 (21.9)	B
8	p.Asp444His	p.Gln456His	Partial	Partial	47.2 (21.7)	B
9	p.Asp444His	p.Gln456His	Partial	Partial	66.3 (19.85)	B
10	p.Asp444His	p.Gln456His	Partial	Partial	45.4 (20.9)	B
11	p.Asp444His	p.Asp444His;p.Ala171Thr	Partial	Partial	52.2 (24.0)	B
12	p.Asp444His	p.Asp444His;p.Ala171Thr	Partial	Partial	55.8 (25.7)	B
13	p.Asp444His	p.Asp444His;p.Ala171Thr	Partial	Partial	44.2 (20.4)	B
14	p.Asp444His	p.Asp444His;p.Ala171Thr	Partial	Partial	53.6 (24.7)	B
15	p.Asp444His	p.Asp444His;p.Ala171Thr	Partial	Partial	54.2 (25.0)	B
16	p.Asp444His	p.Cys33PhefsTer36	Partial	Partial	63.1 (29.1)	B
17	p.Asp444His	p.Cys33PhefsTer36	Partial	Partial	65.1 (30.0)	B
18	p.Asp444His	p.Cys33PhefsTer36	Partial	Partial	48.9 (22.5)	B
19	p.Asp444His	p.Cys33PhefsTer36	Partial	Partial	52.7 (24.3)	B
20	p.Asp444His	p.Cys33PhefsTer36	Partial	Partial	63.5 (29.3)	B
21	p.Asp444His	p.Asp252Gly	Partial	Partial	59.6 (27.5)	B
22	p.Asp444His	p.Asp252Gly	Partial	Partial	53.6 (24.7)	B
23	p.Asp444His	p.Asp444His;p.Thr532Met	Partial	Partial	48.0 (22.1)	B
24	p.Asp444His	p.Arg538Cys	Partial	Partial	64.1 (29.5)	B
25	p.Asp444His	p.Gly114Val	Partial	Partial	44.5 (20.5)	B
26	p.Asp444His	p.Gly34Ser	Partial	Partial	56.0 (25.8)	B
27	p.Asp444His	p.Thr532Met	Partial	Partial	46.7 (21.5)	B
28	p.Asp444His	p.Val62Met	Partial	Partial	57.4 (26.5)	B
29	p.Asp444His	p.Glu436AlafsTer8 *	Partial ^$^	Partial	51.0 (23.5)	B
30	p.Asp444His	p.Gly238AlafsTer36 *	Partial ^$^	Partial	51.7 (23.8)	B
31	p.Asp444His	p.Met98_Ala100delinsIle *	Partial ^$^	Partial	49 (22.6)	B
32	p.Asp444His	p.Asp444His	Mild reduction	Mild reduction	80.6 (37.2)	C
33	p.Asp444His	p.Asp444His	Mild reduction	Mild reduction	68.4 (31.5)	C
34	p.Asp444His	p.Asp444His	Mild reduction	Mild reduction	93.7 (43.2)	C
35	p.Asp444His	p.Asp444His	Mild reduction	Mild reduction	89.5 (41.3)	C
36	p.Asp444His	p.Asp444His	Mild reduction	Mild reduction	102 (47.0)	C
37	p.Asp444His	p.Asp444His	Mild reduction	Mild reduction	101 (46.6)	C
38	p.Asp444His	p.Asp444His	Mild reduction	Mild reduction	78.6 (36.2)	C
39	p.Asp444His	p.Asp444His	Mild reduction	Mild reduction	84.1 (38.8)	C
40	p.Asp444His	p.Asp444His	Mild reduction	Mild reduction	80.0 (37.0)	C
41	p.Asp444His	p.Asp444His	Mild reduction	Mild reduction	89.8 (41.4)	C
42	p.Asp444His	p.Asp444His	Mild reduction	Mild reduction	118.2 (54.5)	C
43	p.Asp444His	p.Asp444His	Mild reduction	Mild reduction	100 (46.1)	C
44	p.Asp444His	p.Asp444His	Mild reduction	Mild reduction	104 (48)	C
45	p.Asp444His	p.Asp444His	Mild reduction	Mild reduction	72.8 (33.6)	C
46	p.Asp444His	p.Asp444His	Mild reduction	Mild reduction	93.7 (43.2)	C
47	p.Asp444His	p.Asp444His	Mild reduction	Mild reduction	80.6 (37.2)	C
48	p.Asp444His	p.Asp444His	Mild reduction	Mild reduction	95.3 (43.9)	C
49	p.Asp444His	p.Asp444His	Mild reduction	Mild reduction	104.8 (48.3)	C
50	p.Asp444His	p.Asp444His	Mild reduction	Mild reduction	97.4 (44.9)	C
51	p.Asp444His	p.Asp444His;p.Ala171Thr	Partial	Mild reduction	80.1 (36.9)	C
52	p.Asp444His	p.Asp444His;p.Ala171Thr	Partial	Mild reduction	76.8 (35.4)	C
53	p.Asp444His	p.Arg157His	Partial	Mild reduction	85.2 (39.3)	C
54	p.Asp444His	p.Pro497Ser	Partial	Mild reduction	69.5 (32.1)	C
55	p.Asp444His	p.Cys33PhefsTer36	Partial	Mild reduction	69.0 (54.1)	C
56	p.Asp444His	p.His323Arg	Partial or Mild reduction	Mild reduction	68.0 (31.3)	C
57	p.Asp444His	p.Pro133Leu *	Partial or Mild reduction	Mild reduction	84.9 (39.2)	C
58	p.Asp444His	p.Pro133Leu *	Partial or Mild reduction	Mild reduction	90.4 (41.7)	C
59	p.Asp444His	p.Pro133Leu *	Partial or Mild reduction	Mild reduction	101 (46.6)	C
60	p.Asp444His	p.Val109Ala *	Partial or Mild reduction	Mild reduction	67.7 (31.2)	C
61	p.Gly293Ser *	p.Phe361Ser *	Unknown	Mild reduction	89.2 (41.1)	C
62	p.His323Arg	p.His323Arg	Partial or Mild reduction	Mild reduction	78.8 (36.3)	C
63	p.His323Arg	p.His323Arg	Partial or Mild reduction	Mild reduction	69.0 (31.8)	C
64	p.Gln456His	WT	Mild reduction	Mild reduction	88.7 (40.9)	C

WT = Wild Type. ^$^ supposed to be due to the deleterious impact of the novel variants. * allelic variants not previously reported in the literature.

**Table 2:** Variants are identified with associated deficit severity. Profound: individuals with a reduction in activity of <10%. Partial: individuals with a reduction in activity of between 10 and 30%. Mild Reduction: Individuals with reduction >30% of mean normal activity.

Variants		Deficit Associated	Reference
Nucleotide Change	Amino Acid Change		
c.98_104delGCGGCTGinsTCC	p.Cys33PhefsTer36	Profound	[21]
c.100G > A	p.Gly34Ser	Profound	[22]
c.184G > A	p.Val62Met	Profound	[23]
c.294_299del	p.Met98_Ale100delinsIle	Unknown	
c.326T > C	p.Val109Ala	Unknown	
c.341G > T	p.Gly114Val	Profound	[24]
c.398C > T	p.Pro133Leu	Unknown	
c.470G > A	p.Arg157His	Profound	[21]
c.511G > A	p.Ala171Thr	profound (allele combined with p.Asp444His)	[20]
c.713delG	p.Gly238AlafsTer36	Unknown	
c.755A > G	p.Asp252Gly	Profound	[25]
c.877G > A	p. Gly293Ser	Unknown	
c.968A > G	p.His323Arg	mild reduction/partial	[15,26,27]
c.1082T > C	p.Phe361Ser	Unknown	
c.1307_1308delAG	p.Glu436AlafsTer8	Unknown	
c.1330G > C	p.Asp444His	partial (in compound heterozygosity with a profound variant)	[28]
c.1368A > T	p.Gln456His	Profound	[28]
c.1489C > T	p.Pro497Ser	Partial	[29]
c.1595C > T	p.Thr532Met	Profound	[28]
c.1612C > T	p.Arg538Cys	Profound	[21]

**Table 3:** Patients with BD: Genotype, enzyme activity at the confirmation, and follow-up. The BTD value stability traffic light system indicates: red = enzyme activity consistently below normal; yellow = fluctuating enzyme activity, sometimes below and sometimes above 30%; green = enzyme activity consistently above 30%.

Patient ID	Allele 1	Allele 2	BTD Enzyme Activity (%) at Confirmation	Age (Months) at BTD Enzyme Activity Recovery	BTD Value Stability	Biotin Treatment at Last Visit	Age at Last Visit (Year)	Outcome/Sequelae
1	p.Gln456His	p.Gln456His	6.92	No	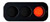	10 mg/day	6	Healthy
12	p.Asp444His	p.Asp444His; p.Ala171Thr	25.75	No	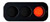	5 mg/day	6	Mild dermatologic signs (hypopigmentation, café-au-lait macule, regressing hemangioma)
27	p.Asp444His	p.Thr532Met	21.55	No	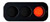	5 mg/day	5.8	Healthy
29	p.Asp444His	p.Glu436AlafsTer8	23.54	3	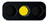	5 mg/day	8.7	Healthy
25	p.Asp444His	p.Gly114Val	20.54	3	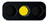	5 mg/day	8.8	Healthy
23	p.Asp444His	p.Asp444His; p.Thr532Met	22.15	3	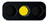	N.A.	N.A.	Follow-up discontinued by patient
24	p.Asp444His	p.Arg538Cys	29.58	3	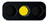	5 mg/day	7.5	Healthy
16	p.Asp444His	p.Cys33PhefsTer36	29.12	12	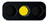	5 mg/day	9	Healthy
31	p.Asp444His	p.Met98_Ala100 delinsIle	22.62	3	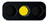	5 mg/day	8,4	Healthy
13	p.Asp444His	p.Asp444His; p.Ala171Thr	20.40	3	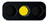	5 mg/day	5.9	Recurrent asthma-like bronchitis
18	p.Asp444His	p.Cys33PhefsTer36	22.57	3	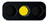	5 mg/day	5.8	Healthy
28	p.Asp444His	p.Val62Met	26.49	3	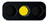	5 mg/day	5.8	Healthy
7	p.Asp444His	p.Gln456His	21.92	3	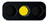	5 mg/day	5	Alternating esotropia
26	p.Asp444His	p.Gly34Ser	25.86	12	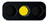	5 mg/day	5.9	Healthy
15	p.Asp444His	p.Asp444His; p.Ala171Thr	25.02	3	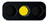	5 mg/day	5.5	Speech therapy for phonetic disorder and language delay
20	p.Asp444His	p.Cys33PhefsTer36	29.31	3	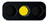	5 mg/day	5.1	Healthy
58	p.Asp444His	p.Gln456His	25.20	36	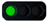	5 mg/day	8	Healthy
21	p.Aps444His	p.Asp252Gly	27.28	3	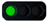	N.A.	N.A.	Follow-up discontinued by patient
22	p.Aps444His	p.Asp252Gly	24.74	3	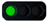	N.A.	N.A.	Follow-up discontinued by patient
11	p.Asp444His	p.Asp444His; p.Ala171Thr	24.0	36	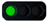	5 mg/day	8	Mild language delay
3	p.Asp444His	p.Gln456His	29.86	60	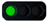	5 mg/day	6.5	Healthy
4	p.Asp444His	p.Gln456His	18.92	3	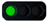	5 mg/day	5.9	Hypertrichosis of the limbs
17	p.Asp444His	p.Cys33PhefsTer36	30	3	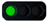	5 mg/day	5.8	Healthy
30	p.Asp444His	p.Gly238AlafsTer36	23.86	60	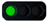	5 mg/day	5.8	Healthy
5	p.Asp444His	p.Gln456His	25.62	3	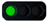	5 mg/day	5.7	Healthy
6	p.Asp444His	p.Gln456His	24.69	36	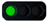	5 mg/day	5	Frequent URTIs; past acute lymphoblastic leukemia
8	p.Asp444His	p.Gln456His	21.78	3	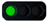	5 mg/day	5	Healthy
14	p.Asp444His	p.Asp444His; p.Ala171Thr	24.74	3	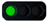	5 mg/day	5	Healthy
9	p.Asp444His	p.Gln456His	19.85	3	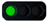	5 mg/day	5.2	Healthy
19	p.Asp444His	p.Cys33PhefsTer36	24.32	36	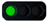	5 mg/day	5.1	Healthy
10	p.Asp444His	p.Gln456His	20.9	3	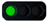	5 mg/day	5.3	Healthy

N.A. = Not Available.

## Data Availability

All clinical data and materials are available in our Pediatric Unit.

## Abbreviations

The following abbreviations are used in this manuscript:

BD	Biotinidase deficiency
BTD	Biotinidase
NBS	Newborn screening
AER	Residual enzymatic activity
DBS	Dried blood spots
VUS	Variant of uncertain significance
ACMG	American College of Medical Genetics and Genomics
HGMD	Human Genetic Mutation Database
WT	Wild Type
RCTs	Randomized controlled trials
