# Correction: Ortolano et al. Enzymatic Evolution and Longitudinal Recovery in Biotinidase Deficiency: Genotypic and Clinical Insights from the Follow-Up of a Newborn-Screened Cohort in Emilia-Romagna, Italy. *Metabolites* 2025, *15*, 605

**DOI:** 10.3390/metabo16030188

**Published:** 2026-03-11

**Authors:** Rita Ortolano, Soara Menabò, Egidio Candela, Giacomo Biasucci, Elisa Bortolamedi, Giulia Montanari, Alessandro Zuccotti, Umberto Cattini, Marcello Lanari, Federico Baronio

**Affiliations:** 1 Pediatric Unit, IRCCS Azienda Ospedaliero-Universitaria di Bologna, 40138 Bologna, Italy; rita.ortolano@aosp.bo.it (R.O.); elisa.bortolamedi2@unibo.it (E.B.); umberto.cattini@aosp.bo.it (U.C.); marcello.lanari@unibo.it (M.L.); federico.baronio@aosp.bo.it (F.B.); 2 Medical Genetics Unit, IRCCS Azienda Ospedaliero-Universitaria di Bologna, 40138 Bologna, Italy; soara.menabo@unibo.it; 3 Department of Medical and Surgical Sciences, Alma Mater Studiorum, University of Bologna, Via Massarenti 11, 40138 Bologna, Italy; 4 Pediatrics and Neonatology Unit, Guglielmo da Saliceto Hospital, 29121 Piacenza, Italy; 5 Department of Medicine and Surgery, University of Parma, 43126 Parma, Italy; 6 Specialty School of Pediatrics, Alma Mater Studiorum, University of Bologna, 40126 Bologna, Italy; giulia.montanari22@studio.unibo.it (G.M.); alessandro.zuccotti@studio.unibo.it (A.Z.)

The authors would like to make the following correction to their published paper [1]. The change is as follows:

## Text Correction

1. 1. Introduction—duplicated epidemiological statement

Two consecutive passages reporting identical epidemiological data and interpretation regarding BD incidence in Tuscany and Umbria are present and should appear only once:

- Another recent Italian study found that the incidence of BD identified by NBS in the regions of Tuscany and Umbria was ten times higher (1:6300) than the global average. This data suggests that the pathology may have often been underdiagnosed, with an incidence detected in retrospective clinical studies being erroneously lower. The high incidence observed in the study is likely related to the high frequency of specific mutations in the Italian population. (Wrong Version—Delete)
- Another recent Italian study reported that the incidence of BD detected through NBS in the regions of Tuscany and Umbria was nearly tenfold higher (1:6300) than the global average. These findings suggest that the disorder has likely been underdiagnosed, as incidence estimates derived from retrospective clinical studies appear to have been erroneously lower. The elevated incidence observed in this study is plausibly attributable to the high prevalence of specific mutations within the Italian population [10]. (Right Version—Keep)

2. Introduction—duplicated statement of study aims

The aims of the study are described twice in immediate succession using near-identical wording.

These paragraphs were previously merged/revised but are now duplicated in the published version:

- The purpose of this study is to provide a critical analysis of the clinical experience regarding BD screening in children born from January 2016 to December 2020 in the Emilia-Romagna Region, with a specific focus on the long-term follow-up of these patients. The incidence of the disease in Emilia-Romagna will be evaluated by comparing it with that described in other national and international settings. The clinical and genetic investigation data obtained from patients and parents will be described. Data from a minimum of 5 years of follow-up for the 31 patients with BD who attend this center will be described. Ultimately, we sought to comprehensively evaluate the age-related trajectory of BTD enzyme activity within our patient cohort. (Wrong Version—Delete)
- The aim of this study is to critically analyze the clinical experience with BD screening in children born between January 2016 and December 2020 in the Emilia-Romagna Region, with a particular emphasis on long-term follow-up. The incidence of the disorder in Emilia-Romagna will be assessed in comparison with that reported in other national and international contexts. Clinical and genetic data from patients and their parents will be presented, together with findings from a minimum of five years of follow-up in 31 BD patients monitored at this center. Finally, we seek to provide a comprehensive evaluation of the age-related trajectory of BTD enzyme activity within this cohort and the need for retesting of patients as recommended by Forny et al. [15]. (Right Version—Keep)

3. Materials and Methods—duplicated description of recall strategy

The recall protocol and classification of enzymatic activity are reported twice with minimal wording differences, despite having been consolidated in the final submitted version:

- All patients recalled from January 2016 to December 2019 for AER < 107 U/dL repeated DBS, and if it returned positive (<107 U/dL), molecular analysis of the BTD gene was performed. In patients recalled from January 2020, molecular analysis of the BTD gene was performed immediately in newborns with a high risk of recall (AER < 65 U/dL) or after the second pathological DBS in newborns with a low risk of recall (AER: 66–85 U/dL). Biotinidase enzymatic activity was expressed either in units per deciliter (U/dL) or as a percentage of mean normal activity. For conversion purposes, reference values were defined as follows: 21.6 U/dL corresponds to 10% of mean normal activity, 65 U/dL corresponds to 30% and 108.4 U/dL corresponds to 50%. The loss of BTD activity was considered profound if <10% of normal activity, partial when between 10 and 30% and mild reduction if between 30 and 50%. (Wrong Version—Delete)
- Between January 2016 and December 2019, all newborns recalled for AER < 107 U/dL underwent repeat DBS testing; if the result remained abnormal (<107 U/dL), molecular analysis of the BTD gene was performed. From January 2020 onward, molecular analysis was conducted directly in newborns at high risk of recall (AER < 65 U/dL) or following a second abnormal DBS in those at lower risk of recall (AER 66–85 U/dL). Biotinidase enzymatic activity was expressed either in units per deciliter (U/dL) or as a percentage of mean normal activity. For conversion purposes, reference values were defined as follows: 21.6 U/dL = 10% of mean normal activity, 65 U/dL = 30%, and 108.4 U/dL = 50%. Loss of BTD activity was classified as profound (<10% of normal activity), partial (10–30%), or mild (30–50%). (Right Version—Keep)

The authors state that the scientific conclusions are unaffected. This correction was approved by the Academic Editor. The original publication has also been updated.
